# Muscle Hypertrophy, Strength, and Salivary Hormone Changes Following 9 Weeks of High- or Low-Load Resistance Training

**DOI:** 10.3390/jfmk11010017

**Published:** 2025-12-30

**Authors:** Marissa L. Bello, Shawn M. Arent, Zachary M. Gillen, JohnEric W. Smith

**Affiliations:** 1 Department of Health and Human Performance, Middle Tennessee State University, Murfreesboro, TN 37132, USA; 2 Department of Exercise Science, University of South Carolina, Columbia, SC 29208, USA; sarent@mailbox.sc.edu; 3 Department of Kinesiology, Mississippi State University, Starkville, Mississippi State, MS 39759, USA; zmg43@msstate.edu (Z.M.G.); johneric.smith@msstate.edu (J.W.S.)

**Keywords:** resistance training, hormones, muscle thickness

## Abstract

**Background:** Resistance training has recently focused more on a high- vs. low-load training approach, suggesting heavier loads optimize strength adaptations through maximal recruitment of motor units, whereas lower loads stimulate a greater hypertrophy response. The purpose of this investigation was to examine and determine significant differences in muscle thickness, strength, and hormonal markers over nine weeks of high- or low-load resistance training. **Methods:** Seventeen recreationally-trained males were recruited for this study (M_age_ = 20.4 ± 2.7 years). Participants were split into training with high-loads (85% 1-RM; *n* = 8) or low-loads (30% 1-RM; *n* = 9) completing 3 whole-body training sessions per week for 9 weeks. Each session included three working sets per exercise of repetitions to failure. Measures were collected at baseline and every three weeks after of muscle thickness (biceps brachii, triceps brachii, pectoral major, rectus femoris, and biceps femoris) and salivary hormones (basal and acute post-exercise testosterone and cortisol). RM-ANOVAs were conducted to analyze changes in hypertrophy and the hormones, with significance set at *p* < 0.05. **Results:** Muscle thickness increased significantly over time for all sites (*p* < 0.05), with no significant group × time interactions except for the triceps brachii (*p* = 0.04). There were no significant changes in basal hormone levels or changes from basal to immediately post exercise (*p* > 0.059). The high-load group showed greater increases in 1-RM following the training program. **Conclusions:** Our results demonstrate similar hypertrophy regardless of training volume and training load, but greater increases in strength in the high-load group. Hormonal data revealed no significant changes in basal cortisol and testosterone, suggesting similar stress and recovery. While nonsignificant for differences pre-post in either marker, the pattern of a slight decrease in testosterone may be an effect of receptor uptake, and additional monitoring over a longer time interval should be used to track the changes over a full recovery window.

## 1. Introduction

Resistance training is a well-established method to improve muscle strength and size. Through progressive overload, hypertrophy of the musculature and greater neuromuscular activation can occur, with a primary focus on increasing the size of the muscle and the strength of contractions and subsequently improving performance in both speed and power.

The hypertrophic and subsequent strength changes in musculature can occur at varying intensities, although a lower repetition, higher load range has traditionally been recommended for strength and a higher repetition, lower load range for inducing greater hypertrophy [1]. The moderate correlation between hypertrophy and strength suggests that both adaptations can occur in parallel, although distinct training stimuli may optimize gains in one outcome relative to the other [2]. Additionally, training variables play an important role as number of repetitions [3], total volume [4], and frequency of the program [5,6] may all influence the magnitude of the adaptations. Meta-analyses suggest that, when volume is equated and sets are performed near failure, hypertrophy occurs across a broad loading spectrum, although a majority of this work has involved untrained participants where the novelty of training elicits rapid growth regardless of load [7,8].

The endocrine response to training provides additional insight into training and recovery status. Testosterone and cortisol are frequently studied as key anabolic and catabolic hormones, respectively, and the responses of these hormones may provide more context with regards to the adaptations that occur with resistance training. Acute increases in testosterone are consistently observed following heavy resistance exercise, with larger responses under higher intensity protocols [9,10]. Cortisol responses are less predictable, often influenced by circadian rhythm and time of day [10,11,12]. For this reason, chronic changes in resting concentrations may better reflect the long-term physiological impact of training.

Longitudinal studies report sustained elevations in testosterone with continued resistance training, whereas detraining returns levels toward baseline [13,14,15]. Cortisol responses to long-term training are inconsistent, with reports of increases [16,17], decreases [18,19], or no change [20]. Variability may be explained by factors such as sex, training status, circadian rhythm, or nutrition, as protein and carbohydrate intake can augment testosterone and blunt cortisol, although findings are mixed [21,22,23,24,25,26]. These observations suggest that resting hormonal changes may provide a more integrated view of training stress and adaptation, but the influence of loading strategies on this response remains unclear.

Recently, a low- versus high-load approach has received increased attention to determine an optimal training stimulus and subsequent adaptations, typically using loads of 30% and >70% 1-repetition max (1-RM) for low- and high-loads, respectively [27]. While heavier loads typically produce greater gains in maximal strength, hypertrophy outcomes appear similar across loading schemes when effort is matched [7,28,29,30,31]. However, existing studies often neglect true strength ranges as defined by the NSCA (≥85% 1-RM) [1] and rarely incorporate endocrine outcomes. Moreover, the reliance on untrained participants limits generalizability to trained populations. Addressing these gaps may provide a more comprehensive view of the resistance training response.

Therefore, the purpose of this study was to examine the effects of nine weeks of high-load (85% 1-RM; HL) and low-load (30% 1-RM; LL) resistance training on muscle hypertrophy and salivary hormones in recreationally trained males. It was hypothesized that the low-load group would demonstrate greater hypertrophy and perturbations in the cortisol response, reflecting the metabolic stress associated with higher repetitions, and the high-load group would demonstrate greater increases in resting testosterone.

## 2. Materials and Methods

### 2.1. Participants

Twenty healthy, resistance-trained males were recruited for this study. Participants were included if they were currently training between 2–4 days per week for at least the previous six months and excluded if they had any medical conditions or injuries that would prevent them from participating. There were three dropouts during the study resulting in 17 participants completing the study. Dropouts were related to scheduling issues (2) or an injury that occurred outside of the study (1). Participant demographics can be found in Table 1. This study was conducted in accordance with the ethical standards of the Helsinki Declaration and was approved by the Institutional Review Board at Mississippi State University.

### 2.2. Experimental Procedures

In week 1 of the study, participants completed informed consent, a physical activity readiness questionnaire (PAR-Q) to screen for any potential medical conditions that may prevent them from participating in this study, a training history questionnaire to screen for training experience and current training habits, and a 3-day dietary recall to establish baseline caloric intake over the course of a typical week. On a separate day, participants completed familiarizations for all exercises and testing procedures. In week 2, and across two days separated by a minimum of 48 h, baseline measures were collected. On the first day, basal salivary cortisol and testosterone were collected immediately upon waking and participants were instructed to place their samples in their freezer until their scheduled session. Muscle thickness was measured via Ultrasound and body composition via bioelectrical impedance analysis. The methodology of these measures is elaborated on below. Testing of muscle thickness and body composition was completed at four timepoints including baseline (T1) and weeks 3, 6, and 9 (T2–T4) of the training program.

Predicted 1-repetition max (1-RM) testing was completed on the second baseline day using a 3–10 repetition max for all movements used in the training intervention. All completed lifts performed by all participants fell within this range. Predicted maxes were used to calculate percentages and loading throughout the training protocol. Max testing procedures were completed according to guidelines by the NSCA [1]. Predicted maxes were assessed at baseline and again at the end of the training program, with these data published previously [32]. Following baseline testing and familiarization, participants were randomized via a computer random number generator into either low- or high-load training (LL vs. HL), where LL (*n* = 9) trained at 30% of 1-RM, and HL (*n* = 8) trained at 85% of 1-RM. Participant loads were adjusted following NSCA guidelines, increasing load by 2.5–5% if a participant could complete a minimum of two repetitions above the target repetition range in their final set in two consecutive training sessions. For HL, this range was six repetitions, and for LL the range was established following the first week of training to establish individual participant ranges. These increases were to ensure participants continued to train at 85% and 30% of 1-RM throughout the study.

Beginning in week 3 of the study, participants completed resistance training three days per week across nine weeks, resulting in 27 training sessions total. To be included in final analysis, participants were required to attend a minimum of 23 sessions (85%), with average compliance of 91.1% for the low-load group and 89.6% for the high-load group. Training sessions were missed or cancelled due to weather patterns that closed campus facilities, holidays, or scheduling conflicts that arose throughout the study. All training sessions were restricted to one participant and two study staff members to follow University-regulated COVID-19 restrictions.

Participants were asked not to train outside of the study and to refrain from any large changes to their diet. If participants were currently taking supplementation of any kind (vitamins, creatine, etc.) for the previous 60 days, they were asked to continue their normal habits. Each participant was at least four hours post-prandial for training and testing sessions. Participants were asked to refrain from the use of caffeine products or preworkout within six hours of their training session. This was done was to maintain a similar training stimulus across all participants and to reduce the impact of a stimulant on the overall training and adaptations. Only three participants reported habitual caffeine or preworkout intake prior to their normal training. To check for compliance throughout the intervention, participants completed a 3-day dietary recall every three weeks during last session of the week. The three days included a training weekday, a non-training weekday, and a weekend day. These days were averaged together to calculate a weekly average and analyzed using dietary software (MyFitnessPal, version 25.0). Dietary variables included absolute calorie intake and absolute gram intake of carbohydrates, fat, and protein. At the end of each training session, participants were provided a post-workout shake consisting of 20 g of ProTYM whey isolate protein (TYM Performance, Dallas, TX, USA) mixed with 475 mL of water.

### 2.3. Training Protocol

This training protocol methodology has been published previously [32]. Briefly, participants arrived for three days of training per week separated by 48 h, with all training sessions occurring at the same time of day. All sessions were under direct supervision of a certified strength and conditioning specialist with a current certification through the NSCA. Two different days were alternated and order of exercises switched between the days. Day 1 order was back squat, deadlift, bench press, T-row, bicep curls, and skullcrushers. Day 2 order was deadlift, squat, T-row, bench press, skullcrushers, and bicep curls. Two warm-up sets were completed prior to working sets with 120 s of rest between all sets. Three working sets were completed for back squat, deadlift, bench press, and T-row, and two working sets were completed for bicep curls and skullcrushers. Training volume-load was calculated as repetitions × sets × weight and is reported elsewhere [32], and progression was calculated on a weekly basis for each lift if a participant could achieve two additional reps on their last working set in two consecutive sessions. To ensure adequate motivation during each session, participants were allowed to create a playlist with songs of their choosing, with the order kept the same between sessions to control for additional extrinsic motivation.

### 2.4. Predicted 1-RM Protocol

All participants completed a predicted 1-RM using a 3–10 repetition max for all movements used in the training intervention. A predicted 1-RM has been shown to be a reliable method of assessing muscular strength and was used to ensure safety of all participants and account for the experience level of the population recruited [23]. All testing procedures were completed according to NSCA guidelines and all completed lifts performed by all participants fell within this range [13]. Predicted 1-RM values were calculated using the Brzycki equation [24]. The predicted maxes were then used to calculate loads for training sessions in each respective group and participant [24]. Each predicted 1-RM began with a warm up with light resistance that easily allowed 5–10 repetitions. After a 2-min rest period, weight was increased between 4–9 kg (10–20 lbs) for upper body movements or 14–18 kg (30–40 lbs) for lower body movements. A second warm up set of 3–5 repetitions was completed at this weight, again followed by a 2-min rest interval. Weight was increased again between 4–9 kg for upper body movements or 14–18 kg for lower body movements. A near-maximal load at estimated 85% of participants perceived 1-RM was used for the third and final set. Participants were instructed to complete as many repetitions as possible with proper exercise technique and motivation from the study team. Testing was completed in the following order: back squat, deadlift, bench press, T-row, bicep curls, skullcrushers. Predicted 1-RM were assessed prior to the training program and again at the end of the training program.

### 2.5. Salivary Collection

Prior to each testing day, participants received their labeled saliva collection vial and instructions for collection. Participants were instructed to complete their saliva collection immediately upon waking using unstimulated passive drool. All participants were instructed to avoid food or caffeine prior to their saliva collection, and to avoid mouth rinse. Participants were told a minimum of 1.0 mL was required for each saliva sample, and all vials were marked prior with the minimum target line. Vials were placed in a −80 °C freezer immediately upon transfer to the study team and for storage until analysis. An additional saliva sample was collected during each testing week (weeks 3, 6 and 9) following completion of the training session. The additional of post-exercise samples resulted in seven collection time points in total (4 basal, 3 post-exercise). Samples were stored in a −80 °C freezer and then thawed for final analysis. Samples were centrifuged at 1500 rcf for 15 min and enzyme immunoassays were performed in duplicate following manufacturer instructions (Salimetrics, Carlsbad, CA, USA) using a Pointe 180 II analyzer (Pointe Scientific Inc, Canton, MI, USA). The sensitivity of the cortisol kit was 0.028 μg/dL, and sensitivity for the testosterone kit was 0.67 pg/mL. Mean intra assay coefficients of variation were 9.4% for cortisol and 8.5% for testosterone for duplicate samples.

### 2.6. College Student Stress Scale

To account for potential external stress factors unrelated to the resistance training intervention, the College Student Stress Scale (CSSS) was used [33]. The CSSS is an 11-point scale used to quantify additional stress factors (coursework, exams, and relationships) external to the training intervention and is particular to the college population used in the current study.

### 2.7. Muscle Thickness Assessment

An ultrasound (LOGIQ e Diagnostic Ultrasound System, General Electric, Wauwatosa, WI, USA) was used to capture images and measure muscle thickness (MT) for the biceps brachii, triceps brachii, pectoralis major, biceps femoris, and rectus femoris. All measurements were performed on the right side of the body and by the same investigator for all participants. A transducer with an imaging frequency bandwidth of 5.0–23.0 MHz and 12.7 × 53 mm footprint was used, and three measurements were taken for each muscle (gain = 58 dB; image depth = 5 cm), with the average used for final analysis. Standardized locations were used to ensure consistency in MT measurements across all participants and timepoints. The triceps brachii was measured at 50% of the distance from the olecranon process to the acromion process along the muscle belly. The biceps brachii was measured directly horizontal from the triceps location. During measurement of the triceps brachii and biceps brachii, participants were seated, relaxed, and resting their arm to the side with their elbow extended at a natural, comfortable position. During measurement of the pectoralis major and rectus femoris, participants were in a supine position on a training table, with their knee relaxed and resting extended on the table. The pectoralis major was measured at the site between the third and fourth rib under the clavicle midpoint. Measurement of the rectus femoris was taken at the anterior midline of the thigh midway between the inguinal fold and the top of the patella. The biceps femoris was measured at midway of the posterior aspect of the fibular head and ischial tuberosity while participants lied prone on the training table.

### 2.8. Data Analysis

All experimental measures are reported as mean ± standard deviation with an alpha level set a priori at *p* < 0.05. Statistical analyses included only participants who completed the full intervention (*n* = 17). Baseline characteristic comparisons between groups were analyzed using an independent samples *t*-test. Basal cortisol, testosterone, CSSS, and dietary intake variables were analyzed using a two-way repeated measures analysis of variance (RM-ANOVA; group × time; 2 × 4). Changes from basal to post-exercise concentrations for the hormones were calculated and analyzed using a two-way RM-ANOVA (group × time; 2 × 3). Absolute and relative changes in predicted 1-RM were analyzed using a one-way ANCOVA with group as a fixed factor and baseline as a covariate. Partial eta squared were calculated for all RM-ANOVAs. Partial eta squared represents the proportion of variance explained by treatment effects and can be useful in interpreting differences. Effects are presented as follows: η_p_^2^: 0.2 = small; η_p_^2^: 0.5 = moderate; η_p_^2^: 0.8 = large. All statistical analyses were completed using SPSS (Version 30, IBM Corporation, Armonk, NY, USA).

## 3. Results

There were no significant differences in the CSSS across time (*p* = 0.657), between groups (*p* = 0.373), and no significant group × time interaction (*p* = 0.638). Dietary intake data has been reported previously [32], with no significant differences across time for all variable (*p* > 0.374), no significant differences between groups (*p* > 0.098), and no significant group × time interactions observed (*p* > 0.061).

### 3.1. Cortisol

**Figure 1:**
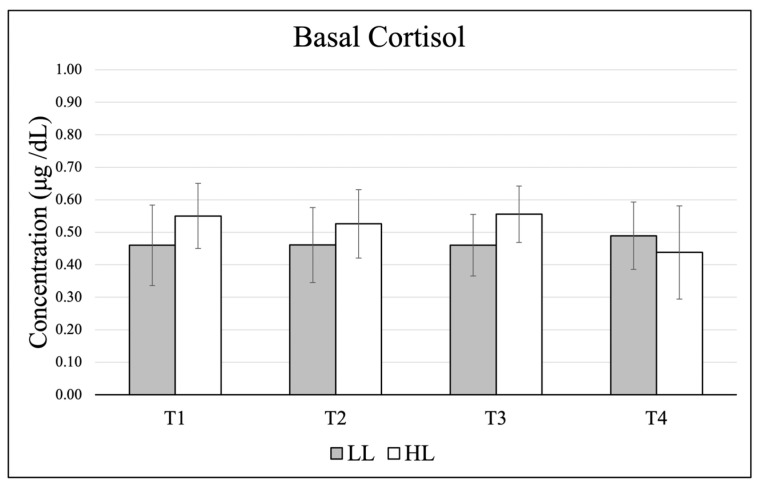
Changes in basal cortisol over the course of the training intervention.

**Figure 2:**
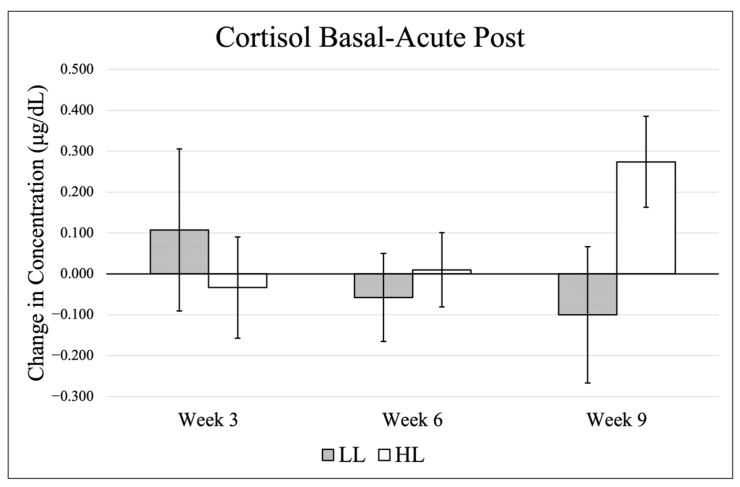
Cortisol differences from basal to post-exercise.

Data (mean ± SE) for basal cortisol are presented in Figure 1, and changes from basal to post-exercise are shown in Figure 2. There were no significant effects in basal levels across time (*p* = 0.929, η_p_^2^ = 0.033), between groups (*p* = 0.693, η_p_^2^ = 0.011) and no significant group × time interaction (*p* = 0.739, η_p_^2^ = 0.089). When comparing change scores (acute post-exercise–basal value) for each testing timepoint, there was no significant time effect (*p* = 0.659, η_p_^2^ = 0.058), group effect (*p* = 0.479, η_p_^2^ = 0.034), or group × time interaction (*p* = 0.154, η_p_^2^ = 0.235).

### 3.2. Testosterone

**Figure 3:**
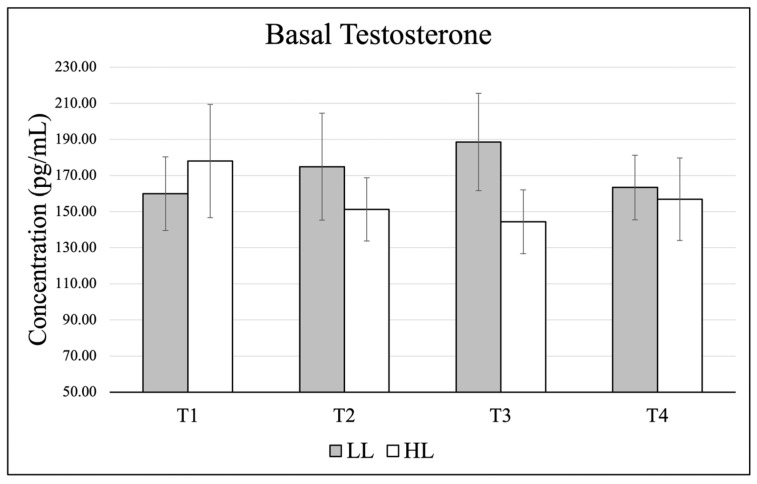
Changes in basal testosterone over the course of the training intervention.

**Figure 4:**
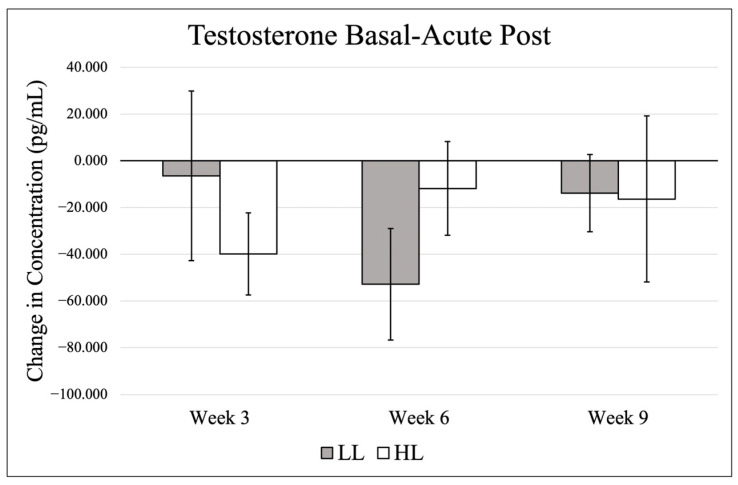
Testosterone differences from basal to post-exercise.

Data (mean ± SE) for basal testosterone are presented in Figure 3, and changes from basal to post-exercise are shown in Figure 4. There were no significant effects in basal levels for time (*p* = 0.894, η_p_^2^ = 0.044), group (*p* = 0.612, η_p_^2^ = 0.018), and no significant group × time interaction (*p* = 0.394, η_p_^2^ = 0.199). When comparing change scores for each testing timepoint (acute post-exercise–basal value), there was no significant time effect (*p* = 0.551, η_p_^2^ = 0.082), group effect (*p* = 0.952, η_p_^2^ = 0.000). or group × time interaction (*p* = 0.059, η_p_^2^ = 0.332).

### 3.3. Predicted 1-RM

These data are reported elsewhere [32]. Briefly, after adjustments for baseline, there were no statistically significant group differences in absolute predicted 1-RM (*p* > 0.179). Significant group differences in relative predicted 1-RM were observed in squat (*p* = 0.011, η_p_^2^ = 0.362), deadlift (*p* = 0.049, η_p_^2^ = 0.235), and bicep curl (*p* = 0.006, η_p_^2^ = 0.409), with the HL group demonstrating greater increases compared to LL. There were no relative differences between groups for bench, T-row, or skullcrushers (*p* > 0.106).

### 3.4. Muscle Thickness

Muscle thickness data for all muscles are presented in Table 2. A significant time effect was observed for biceps brachii MT (*p <* 0.001, η_p_^2^ = 0.363), with pairwise comparisons indicating increases from T1 to T3 (*p* = 0.012) and T4 (*p* < 0.001). There were no significant group differences (*p* = 0.689, η_p_^2^ = 0.103) or a group × time interaction (*p* = 0.542, η_p_^2^ = 0.046).

Triceps brachii also demonstrated a significant time effect (*p* = 0.002, η_p_^2^ = 0.278), with a significant increase from T1 to T4 (*p* = 0.009). In addition, a significant group × time interaction (*p* = 0.007, η_p_^2^ = 0.236) was observed. Post hoc analyses revealed the 85% group exhibited significant increases from T1 to T2 (*p* = 0.026) that remained increased at T3 (*p* = 0.003) and T4 (*p* = 0.003). No significant groups differences were found (*p* = 0.103, η_p_^2^ = 0.168).

There were no significant differences in muscle thickness of the pectoralis major across time (*p* = 0.823, η_p_^2^ = 0.020), group (*p* = 0.092, η_p_^2^ = 0.178), or a group × time interaction (*p* = 0.342, η_p_^2^ = 0.220).

The biceps femoris demonstrated a significant time effect (*p* = 0.028, η_p_^2^ = 0.181), with pairwise comparisons showing increases from T1 to T2 (*p* = 0.020), T3 (*p* = 0.049), and T4 (*p* = 0.036). There were no significant group differences (*p* = 0.318, η_p_^2^ = 0.066), or a group × time interaction (*p* = 0.183, η_p_^2^ = 0.101).

Rectus femoris MT showed a significant time effect (*p* = 0.002, η_p_^2^ = 0.272), with increases from T1 to T2 (*p* = 0.003) and T1 to T3 (*p* = 0.016), before decreasing from T3 to T4 (*p* = 0.011). There were no group differences (*p* = 0.110, η_p_^2^ = 0.162) or a group × time interaction (*p* = 0.505, η_p_^2^ = 0.050).

## 4. Discussion

This study examined hypertrophic and hormonal adaptations to nine weeks of high- and low-load resistance training to volitional fatigue. Contrary to our hypotheses, neither basal nor changes in post-exercise concentrations of cortisol or testosterone changed significantly across time or between groups. However, both protocols produced significant increases in muscle thickness, with only the triceps brachii showing a group × time interaction favoring high-load training.

The lack of hormonal differences aligns with previous work indicating that resting or acute endocrine markers do not necessarily predict hypertrophic outcomes [34]. Hormonal responses are transient and highly influenced by factors such as sampling time, circadian rhythm, and prior training stress. The lack of significance in the change from basal levels to immediately post exercise may be largely attributed to scheduling and timing of sessions. Out of nine participants in the LL group, six had morning sessions, while only two out of eight participants in the HL group were in the morning. While the participants were randomized to their intervention, this may impact the interpretation of the cortisol results. Cortisol peaks immediately upon waking [15], and the concentrations and/or secretion rate may have remained elevated despite an appropriate stimulus presented in the earlier training sessions. While the individual timing of sessions may have impacted the hormonal results more than any other performance measure, having each participant train at the same time each session minimized diurnal variations [24,35,36]. Further, cortisol peaks post-exercise around 30-min, while the current study required participants to provide a sample immediately-post training. Thus, the cortisol values may be understated by missing the peak window.

There were no large fluctuations in the HL group for basal cortisol, and while not significant, the LL group presented a pattern of decreasing cortisol over time, particularly in the final resting timepoint and the final post-exercise sample. Interestingly, the patterns of cortisol changes from basal to post between the two groups were inverse. At T1, the HL group showed decreases and the LL group demonstrated increases, whereas this pattern was reversed by T3. By the conclusion of the training intervention, these responses approached statistical significance (*p* = 0.076). This pattern may indicate an initial novelty effect in the LL group, as most participants in the current study had limited prior exposure to low-load training and/or training to failure. Incorporating a pre-exercise sample collected immediately before training sessions would improve characterization of true within-day cortisol responses, particularly for participants training later in the day.

Testosterone has been implicated in the regulation of post-exercise muscle signaling pathways [37], and prior work has shown that low-load, high-volume resistance training (30% 1-RM) can elicit greater muscle protein synthesis compared with high-load, low-volume protocols (90% 1-RM) following unilateral leg extension exercise [27]. In the present study, both groups exhibited a modest reduction in testosterone following training sessions. Although these changes did not reach statistical significance, the observed pattern is noteworthy, as most resistance exercise studies report acute elevations in testosterone following exercise [9,10]. However, acute reductions in testosterone have been documented previously, particularly following resistance training to failure [38]. One potential explanation is increased androgen receptor uptake, as the current study measured free testosterone via saliva, and resistance training is known to upregulate androgen receptor signaling [39,40]. Enhanced receptor binding following exercise could transiently decrease circulating free testosterone. It is also possible that testosterone concentrations rebound during recovery, as previous research demonstrated a return toward baseline or elevated levels within 24 h post-exercise [38].

Significant increases in muscle thickness were observed in all muscles except the pectoralis major, with no overall group differences. These findings are consistent with prior research indicating that when training is performed to failure, both high- and low-load resistance exercise can elicit similar hypertrophic adaptations [34,41,42]. The only significant group × time interaction occurred in the triceps brachii, where hypertrophy occurred earlier and more consistently in the HL group. Similarly, biceps brachii thickness increased significantly in both groups by week nine, though gains occurred sooner in the high-load condition. These findings may suggest a slightly accelerated hypertrophic response with heavier loads, possibly reflecting greater recruitment of high-threshold motor units earlier in the training process. However, the overall similarity in muscle thickness across loads supports the notion that total training volume and effort to failure are primary drivers of hypertrophy, rather than load alone. This may be particularly true when the low-load training represents a novel stimulus. The lack of pectoralis major hypertrophy could be attributed to interindividual differences in technique, fiber activation patterns during pressing exercises, or differences in regional gain within the muscle.

In line with previous work, our findings indicate that hypertrophy adaptations were similar between HL and LL resistance training when sets were taken to failure. However, integrating the predicted 1-RM outcomes provides important context for interpreting these results. Despite comparable increases in muscle thickness across nearly all sites, the HL group demonstrated greater improvements in relative strength for the squat, deadlift, and biceps curl. This difference between hypertrophy and strength reinforces the established principle that heavier loads more effectively drive neural and mechanical adaptations specific to maximal force production. Importantly, this occurred despite no meaningful differences in hormonal responses over the training period. Rather than driving contrary adaptations, the hormonal data suggest that both loading schemes created a relatively comparable internal environment, and that the observed differences in predicted 1-RM were primarily due to the mechanical characteristics of the training itself.

### 4.1. Limitations and Implications for Future Research

While the use of a 3-day recall for nutritional intake can be a valid measure, it still relies on a participant’s recall abilities and may under or overestimate their intake. Given the potential influence of diet on hormonal responses to exercise [23,25,43], both overall dietary intake and meal timing relative to training sessions were monitored. The absence of meaningful changes in reported intake across recalls suggests that participants maintained consistent dietary habits throughout the intervention. While assessments for how participants were currently training or current volume was not tracked, participants reported resistance training habits of an average of three days per week using typical resistance training modalities (free weights, barbells, etc.). This may impact the findings as participants may have been exposed to significant changes in training volume or a novel stimulus. Participants were instructed to refrain from engaging in any additional structured exercise outside of the study protocol. While unplanned activity could contribute to accumulated fatigue and potentially influence training performance, compliance was monitored by asking participants before each session regarding any additional physical activity. To account for the impact of external stress [44,45], the use of the CSSS was used to assess for outside stressors the participants may experience. There were no significant changes over the course of the training program, suggesting stress levels were consistent and likely had minimal influence on the resulting cortisol values. The lack of significance and interpretation of the hormone data in the current investigation suggests the sample size may be need to be increased to further distinguish significant results, and may not translate from males to females, as they do not necessarily respond the same [22], and should therefore be interpreted within the context of a male-only sample, with future research including female participants. Finally, participants in the present study were recreationally trained, which limits generalizability to both novice individuals and highly trained athletic populations. Future investigations should include these groups, as novice lifters may exhibit larger training-induced adaptations, while experienced lifters may respond differently to novel loading strategies.

### 4.2. Conclusions

In conclusion, nine weeks of high- or low-load training to failure produced similar hypertrophy without significant endocrine differences. The absence of significant hormonal changes despite measurable hypertrophy suggests local muscular signaling, rather than systemic hormone fluctuations, primarily mediates adaptation. Stable testosterone and cortisol concentrations likely reflect adequate recovery and adaptation to training load. These findings reinforce that muscle growth can occur independently of systemic hormonal changes and that both heavy and light loads can effectively promote adaptation when effort is maximized.

## Figures and Tables

**Table 1:** Participant Demographics.

Characteristic	30% (LL, *n* = 9)	85% (HL, *n* = 8)
Age (years)	20.3 ± 1.6	20.5 ± 3.7
Weight (kg)	85.4 ± 14.6	69.4 ± 15.0
Height (cm)	176.39 ± 9.34	173.99 ± 6.22
Years Training (yrs)	2.7 ± 1.1	2.0 ± 1.2
Sessions Missed	2.4 ± 0.7	2.8 ± 1.0

Data are presented as mean ± SD.

**Table 2:** Changes in muscle thickness.

Muscle	Group	T1	T2	T3	T4
Biceps (cm)	Overall	3.16 ± 0.70	3.27 ± 0.57	3.35 ± 0.68 *	3.46 ± 0.58 *
	85%	2.95 ± 0.64	3.13 ± 0.63	3.23 ± 0.68	3.34 ± 0.56
	30%	3.35 ± 0.73	3.39 ± 0.51	3.47 ± 0.70	3.58 ± 0.61
Triceps (cm)	Overall	3.37 ± 0.92	3.43 ± 0.84	3.51 ± 0.81	3.62 ± 0.85 *
	85%	2.88 ± 0.76	3.08 ± 0.64 *	3.26 ± 0.75 *	3.32 ± 0.69 *
	30%	3.81 ± 0.85	3.75 ± 0.91	3.74 ± 0.83	3.89 ± 0.92
Chest (cm)	Overall	2.09 ± 0.71	2.11 ± 0.70	2.11 ± 0.56	2.05 ± 0.54
	85%	1.75 ± 0.39	1.85 ± 0.43	1.84 ± 0.44	1.85 ± 0.39
	30%	2.39 ± 0.82	2.34 ± 0.83	2.34 ± 0.57	2.23 ± 0.61
Hamstrings (cm)	Overall	3.20 ± 0.52	3.33 ± 0.48 *	3.38 ± 0.46 *	3.42 ± 0.50 *
	85%	3.02 ± 0.42	3.20 ± 0.42	3.36 ± 0.43 *	3.27 ± 0.43
	30%	3.37 ± 0.61	3.44 ± 0.52	3.39 ± 0.51	3.55 ± 0.56
Quadriceps (cm)	Overall	2.23 ± 0.44	2.39 ± 0.47 *	2.37 ± 0.40 *	2.25 ± 0.44
	85%	2.07 ± 0.37	2.18 ± 0.41	2.23 ± 0.44	2.07 ± 0.47
	30%	2.37 ± 0.48	2.58 ± 0.45	2.49 ± 0.34	2.42 ± 0.36

Data are presented as mean ± SD. * Significantly different from T1 (*p* < 0.05).

## Data Availability

The original contributions presented in this study are included in the article. Further inquiries can be directed to the corresponding author.

## Abbreviations

The following abbreviations are used in this manuscript:

HL	High-load (85% 1-RM) group
LL	Low-load (30% 1-RM) group
